# Analysis of acute non-pharmaceutical toxic exposures in children: a 5-year retrospective study

**DOI:** 10.3389/fpubh.2025.1510205

**Published:** 2025-02-05

**Authors:** Yanning Qu, Rui Tang, Zhuyan Duan, Mengyi Sheng, Hui Wang, Shuang Liu, Jiao Li, Linlin Guo, Linying Guo, Si Zheng

**Affiliations:** ^1^Department of Critical Care Medicine, Children's Hospital Affiliated to Capital Institute Pediatrics, Beijing, China; ^2^Institute of Medical Information, Chinese Academy of Medical Sciences and Peking Union Medical College, Beijing, China; ^3^Information Department, Children's Hospital Affiliated to Capital Institute Pediatrics, Beijing, China; ^4^Department of Computer Science and Technology, Institute for Artificial Intelligence, Tsinghua University, Beijing, China

**Keywords:** acute non-pharmaceutical toxic exposure, children, poisoning, characteristics, outcomes

## Abstract

**Objective:**

This study aims to systematically analyze the epidemiological characteristics, clinical interventions and outcomes of children with acute non-pharmaceutical toxic exposures.

**Methods:**

This retrospective study included all children with acute non-pharmaceutical toxic exposure admitted to the emergency department of the Capital Institute of Pediatrics between January 1, 2019, and December 31, 2023. Eligible patients were triaged into red, yellow, and green groups according to their severity condition. Clinical information including demographics, exposure details, clinical manifestation, laboratory results, treatments, and outcomes were extracted from electronic medical records. Univariate and multivariate logistic regression analyses were conducted to identify risk factors associated with hospitalization.

**Results:**

Overall, a total of 718 patients with acute non-pharmaceutical toxic exposures was included in this study, infants and toddlers accounting for 57.9%. The male-to-female ratio was 1.16:1. The majority exposure events occurred at home (89.3%) and in urban settings (78.4%). Accidental poisoning was the predominant cause, accounting for 94.7%, and the primary route of exposure was oral (93.6%). Mercury was the most common exposed substance, representing 18.8% of cases, particularly among preschool-aged children (31.7%). Patients triaged to red zone had a higher proportion of clinical manifestation and required more aggressive treatments. Although most patients discharged without treatment (78.4%), 19.1% need emergency observation, and 2.5% required hospitalization. Logistic regression analysis showed that corrosive household products exposure (OR = 42.747, 95% CI[5.041–362.520]), skin and mucosal damage (OR = 37.052, 95% CI[5.339–257.153]), pesticides exposure (OR = 33.322, 95% CI[3.863–287.423]), heavy metals exposure (OR = 31.636, 95% CI[1.471–680.210]), neurological manifestation (OR = 22.656, 95% CI[4.766–107.711]), positive toxicology results (OR = 15.105, 95% CI[6.584–34.656]), industrial products exposure (OR = 10.294, 95% CI[1.144–92.641]), and intentional poisoning (OR = 3.060, 95% CI[1.733–5.405]) associated with hospitalization.

**Conclusion:**

Pediatric patients exposed to some specific toxicants like industrial products and corrosive household products might associated with a higher risk of severe clinical outcomes. Advocating for enhanced safety regulations or educations and public health initiatives to mitigate the incidence of such exposures is still important for protecting children's health.

## 1 Introduction

Acute poisoning is one of the most common and serious health issues encountered in pediatric emergency departments (PEDs). According to data from the World Health Organization (WHO), it is estimated that 45,000 children and adolescents died from acute poisoning annually (1). A recent study showed that the incidence of pediatric acute poisoning has been rising annually in China in recent years, posing a significant threat to child health (2). Common causes of pediatric acute toxic exposures include contact with chemicals, inhalation of toxic gases, and ingestion of contaminated food. Pediatric acute toxic exposure is categorized into two major classes: pharmaceutical exposure and non-pharmaceutical exposure. Due to the behavioral tendencies of children, they are particularly vulnerable to various non-pharmaceutical toxicants, including pesticides, industrial chemicals, cleaning agents, and other household products (3, 4). These non-pharmaceutical exposures can lead to serious health consequences for children, and in some cases even, be life-threatening.

The distribution of non-pharmaceutical toxic exposure events in children varied greatly in age, gender, exposure time, exposure region and active contact reason. Meanwhile, changes in the types of toxicants available in the market over time, even within the same region, various studies have focused on acute poisoning in children across different regions of China (5–9) and indicated diverse poisoning case characteristic. At present, widespread availability of household products and inadequate storage measures, especially in settings with insufficient supervision (10) lead to accidental poisoning in children, and the accessibility and hazardous nature of non-pharmaceutical substances make them a common choice for suicide among adolescents (11). Thus continuous monitoring remains crucial for identifying specific trends in pediatric toxic exposures to recognize and manage such poisoning events effectively.

This study aims to summarize the general characteristics, therapeutic presentations, clinical interventions, and outcomes of children with acute non-pharmaceutical toxic exposures through a 5-year retrospective analysis at a single pediatric center. Through this research, we seek to comprehensively understand the epidemiological features of pediatric acute non-pharmaceutical exposures, identify key factors influencing patient hospitalization, and propose relevant preventive and intervention measures to reduce the harm of poisonings in children.

## 2 Methods

### 2.1 Study population

This retrospective study involved all children presented to the emergency department of the Capital Institute of Pediatrics (CIP) for acute non-pharmaceutical toxic exposures from January 1, 2019, to December 31, 2023. The inclusion criteria were as follows: (1) children aged 0 to 18 years; (2) complete clinical data records; (3) visit due to acute non-pharmaceutical toxic exposure. The exclusion criteria were: (1) Cases of drug exposure, including any exposure due to accidental ingestion, overdose, or contact with drugs, such as prescription drugs, over-the-counter drugs, herbal medicines, etc.; (2) Cases of chronic toxic exposure, referring to chronic exposure to low doses of toxins over a long period, such as occupational exposure or environmental pollution; (3) Cases of poisoning after contact with animals including poisoning caused by animal bites, stings, or contact with secretions, such as snake bites, insect bites, or venom gland secretions; (4) For patients who visit multiple times due to the same toxic exposure, only the first visit record will be retained to eliminate bias from multiple records of a single exposure and maintain the validity of the assumptions required for subsequent statistical analyses; (5) Cases where sufficient medical record information cannot be obtained. Totally, 718 eligible children were included. The Ethics Committee of the Capital Institute of Pediatrics approved this study (SHERLLM2024030).

### 2.2 Data processing and variable extraction

The anonymous clinical records for the eligible patients were collected. The extracted variables included age, gender, place of residence, exposure place, time interval from exposure to hospital admission, exposure time, exposure season, route of exposure (oral, dermal, inhalation), exposure reason, caregivers during exposure, caregivers' education level, type of toxicant, clinical manifestations after exposure, laboratory test results, treatment methods, and outcomes.

In this study, toxicants were categorized as follows: mercury, pesticides (insecticides, cockroach poison, rodenticides, etc.), corrosive household cleaning products (disinfectants, toilet cleaners, strong acids, strong alkalis, etc.), non-corrosive household cleaning products (soap, hand wash, laundry detergent, etc.), industrial chemicals (fuels such as gasoline and kerosene, cyanides, benzene, solvents, etc.), desiccants, cosmetics, alcohol-containing products, paints and inks (ink, pigments, pen inks, etc.), plants/mushrooms, contaminated food (contamination by toxic but not for bacterial contamination), heavy metals, and inhaled toxic gases (carbon monoxide, hydrogen sulfide, phosphine, etc.).

All children were grouped into different age groups: infancy and toddler (age < 3 years), preschool age (≥3 years and < 6 years), school age (≥6 years and < 12 years), and adolescence (≥12 years and < 18 years).

Upon arrival at the emergency department, children were triaged based on the severity of their conditions (Table 1):

**Table 1:** Triage strategy for poisoning case in our PEDs.

	**Red zone**	**Yellow zone**	**Green zone**
Symptom	Unstable vital signs or life-threatening conditions	Temporarily stable vital signs but at risk of potential life-threatening conditions	Mild symptoms or chronic conditions
Therapeutic intervention	Requiring immediate resuscitation and intervention within 10 min	Require treatment within 30 min	Delayed treatment would not significantly affect their prognosis
Poisoning Severity Score (12)	Fatal	Moderate to severe	None to minor

### 2.3 Toxicological analyses

In this study, toxic substance detection in blood and urine samples was conducted using a gas chromatography-mass spectrometry (GC-MS) system (GC-MS-QP2020 NX, Shimadzu Corporation, Japan) for both qualitative and quantitative analysis. Chromatographic separation was performed on a DB-5MS capillary column (30 m × 0.25 mm, 0.25 μm). The carrier gas was high-purity helium (>99.999%) with a column flow rate of 1.2 mL/min.

The injection method was splitless, with an injection volume of 1 μL. The column temperature program started at an initial temperature of 50°C (held for 4 min), followed by an increase to 300°C at a rate of 20°C/min, and held at 300°C for 20 min, resulting in a total run time of 36 min.

Mass spectrometry detection was performed using an electron ionization (EI) source with an ionization energy of 70 eV. The ion source temperature was set at 230°C, the interface temperature at 250°C, and the mass range for full scan mode was set to m/z 50–500.

During the detection process, samples underwent pre-treatment before gas chromatographic separation and mass spectrometry detection. The qualitative and quantitative analysis of toxins was accomplished by comparing the obtained results with those of standard reference substances.

### 2.4 Statistical analysis

We used SPSS 26.0 (IBM Corp., Armonk, N.Y., USA) and Python 3.11 for statistical analysis. Categorical data were measured with frequencies and percentages, and differences between groups were analyzed using the chi-square test or fisher's exact test. We employed pairwise comparison to analyze differences across age or triage groups. For each exposure in a specific age or triage group, a 4-fold table was constructed comparing this group with others. Fisher's exact test was applied when more than 20% of the expected frequencies were < 5. We used univariate and multivariate logistic regression to identify the risk factors associated with hospitalization of acute pharmaceutical toxic exposure. *P* < 0.05 was considered significant for statistical test.

## 3 Results

### 3.1 Basic characteristics

From January 1, 2019, through December 31, 2023, a total of 718 children presented to the emergency department due to acute non-pharmaceutical toxic exposures. The basic characteristics are detailed in Table 2. The age range of the patients was 0–16 years, with a mean age of 3.8 ± 3.4 years and more than half of the cases were infants and toddlers (57.9%, *n* = 416). The male-to-female ratio was 1.16:1, with a slightly higher proportion of males compared to females (Supplementary Table S1). Most exposures occurred in urban areas (78.4%, *n* = 563) and at home (89.3%, *n* = 641). The median time from exposure to hospital presentation was 3 h, with 73.1% of patients (*n* = 525) arrived at the hospital within 4 h after exposure (as gastric lavage can be considered as a treatment option for children arriving within 4 h). The exposure time was primarily concentrated between 16:00 and 24:00 (52.1%, *n* = 374), with a relatively higher incidence in the autumn season (30.6%, *n* = 220). Oral ingestion route was noted in 93.6% of cases (*n* = 672), with a smaller proportion resulting from skin contact (1.3%, *n* = 9) and respiratory inhalation (5.2%, *n* = 37). As for the causes of exposure, accidental exposure was the most common, accounting for 94.7% (*n* = 680). Intentional exposure was reported in only 38 cases involving children aged 10–16 years. Of these 76.3% were girls and 23.7% boys. Notably, 64.9% of the children with intentional exposure were associated with a diagnosis of depression.

**Table 2:** Basic characteristics of children with acute non-pharmaceutical toxic exposures.

**Basic characteristics**	***N*(%)**
**Gender**	
Male	385(53.6%)
Female	333(46.4%)
**Age**	
Infant and Toddler (< 3 years)	416(57.9%)
Preschool age (≥3 years age and < 6 years)	167(23.3%)
School age (≥6 years age and < 12 years)	93(13.0%)
Adolescence (≥12 years age and < 18 years)	42(5.8%)
**Residence**	
Urban	563(78.4%)
Rural	155(21.6%)
**Exposure place**	
Home	641(89.3%)
Others	77(10.7%)
**Time interval from exposure to hospital admission**	
≤ 4 h	525(73.1%)
>4 h	193(26.9%)
**Exposure time**	
8:00–16:00	281(39.1%)
16:00–24:00	374(52.1%)
00:00–8:00	63(8.8%)
**Route of exposure**	
Oral	672(93.6%)
Dermal	9(1.3%)
Inhalation	37(5.2%)
**Exposure reason**	
Accidental	680(94.7%)
Intentional	38(5.3%)
**Caregiver during exposure**	
Parents	369(51.4%)
Grandparents	184(25.6%)
None	133(18.5%)
Others	32(4.5%)
**Educational level of caregiver**	
High school or below	136(18.9%)
College/University	364(50.7%)
Postgraduate or above	162(22.6%)
Unknown	56(7.8%)

**Figure 1:**
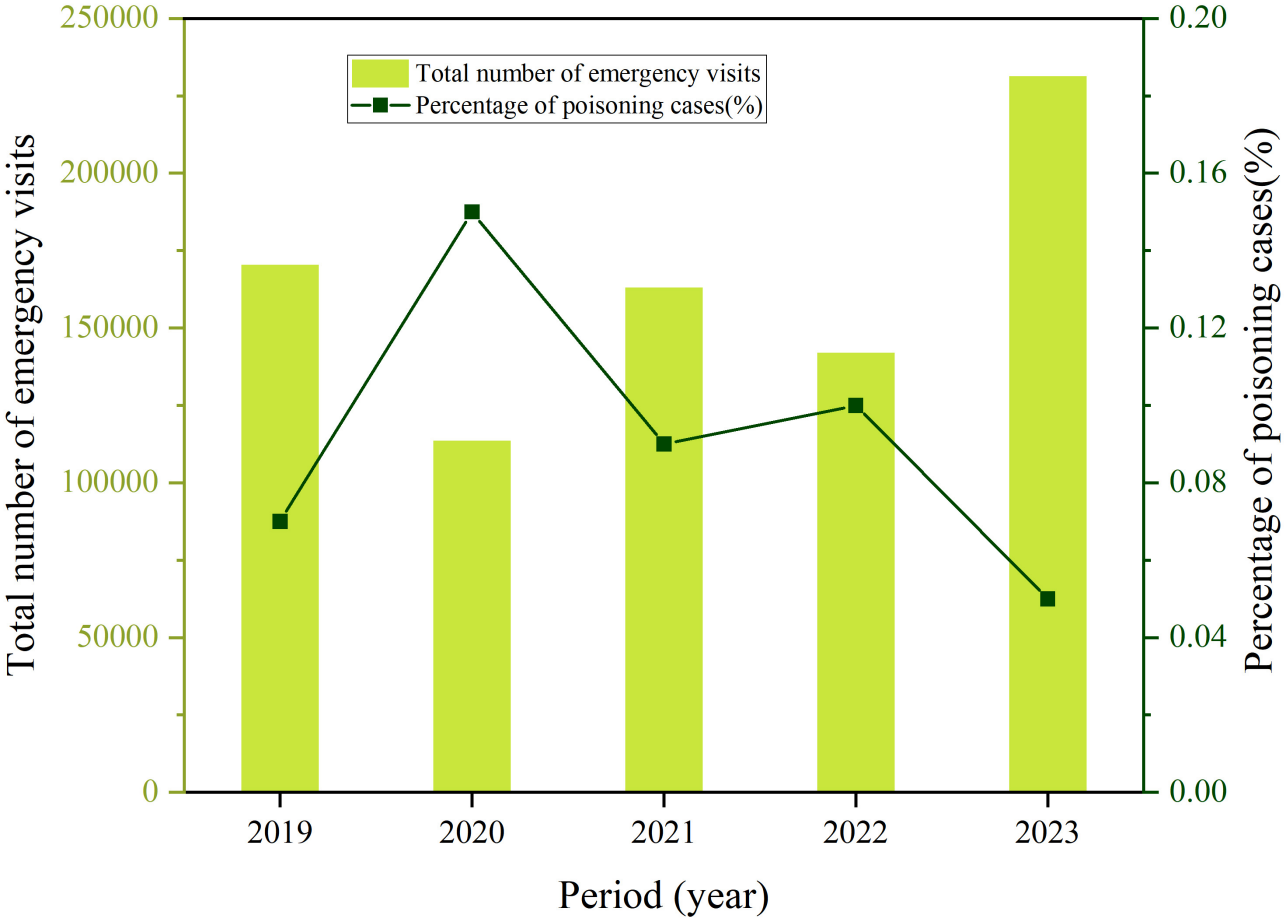
The annual total number of emergency visits and proportion of pediatric patients with acute non-pharmaceutical exposure.

Over the past 5 years, the annual number of children presenting to the emergency department due to non-pharmaceutical toxic exposures fluctuated between 125 and 169 cases. The proportion of these cases relative to the total annual visits is shown in Figure 1, with percentages ranging from 0.05% to 0.15%. Notably, the total visits and proportion of children with non-pharmaceutical toxic exposures reached to the peak in 2020.

### 3.2 Exposure causes

**Figure 2:**
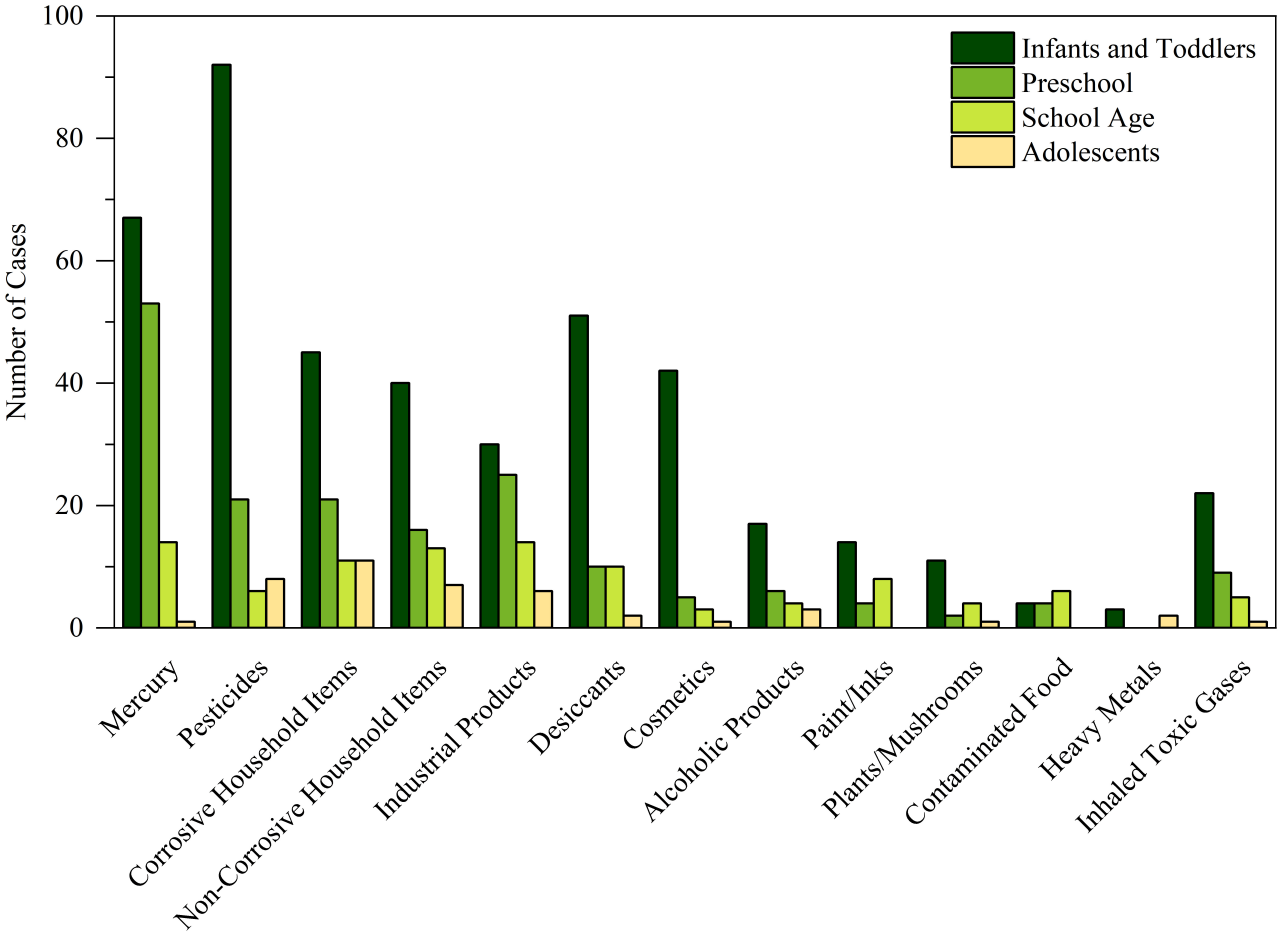
The distribution of non-pharmaceutical toxic substance across children indifferent age groups.

The distribution of different types of non-pharmaceutical toxic exposures varied across different age groups (Figure 2, Supplementary Table S2), more details of exposure causes in different poisoning category is provided in Supplementary Tables S3, S4. Among the different types of toxicants, mercury was the most common substance, accounting for 18.8% of the total cases (*n* = 135), followed by pesticides (17.7%, *n* = 127) and corrosive household products (12.3%, *n* = 88). Preschool children were the main population of mercury exposure (31.7%) (*P* < 0.05), while pesticides exposure was more likely to occur in infants and toddlers (22.1%). The occurrence of corrosive household products exposure was higher among adolescents compared to other groups. Industrial chemicals accounted for 10.4% of exposures, with similar rates in preschool-aged children, school-aged children, and adolescents, while the rate was slightly lower in infants and toddlers. Desiccants exposures constituted 10.2% of cases (*n* = 73), with a slightly higher proportion in infants and toddlers, while cosmetic exposures (7.1%, *n* = 51) were most common among infants and toddlers. No significant differences observed between each age groups for non-corrosive household products, alcoholic products, plants/mushrooms and inhaled toxic gases (*P* > 0.05) (Supplementary Table S5).

### 3.3 Clinical manifestations

77.6% of the children with acute non-pharmaceutical exposure showed no clinical manifestations (*n* = 557). Besides, the proportions of patients with gastrointestinal system (42.4%), skin and mucous membrane (13.0%), nervous system (16.3%), circulatory system (14.1%), hematologic system (8.7%) and urinary (6.5%) manifestation were significantly higher in the red zone (both *P* < 0.05). Similarly, multi-system involvement (28.3%) was significantly more frequent in the red zone, indicating more severe clinical presentations in these patients (Table 3, Supplementary Table S6), more detail about clinical manifestation is provided in Supplementary Table S7.

**Table 3:** Clinical manifestations of children with acute non-pharmaceutical toxic exposures.

**Clinical manifestation**	**Total (*n =* 718)**	**Red zone (*n =* 92)**	**Yellow zone (*n =* 463)**	**Green zone (*n =* 163)**
No manifestation	557(77.6%)	32(34.8%)	375(81.0%)	150(92.0%)
Gastrointestinal system	140(19.5%)	39(42.4%)	88(19.0%)	13(8.0%)
Skin or mucous membrane	19(2.6%)	12(13.0%)	6(1.3%)	1(0.6%)
Respiratory system	16(2.2%)	5(5.4%)	11(2.4%)	0(0%)
Nervous system	32(4.5%)	15(16.3%)	17(3.7%)	0(0%)
Circulatory system	17(2.4%)	13(14.1%)	4(0.9%)	0(0%)
Hematologic system	14(1.9%)	8(8.7%)	6(1.3%)	0(0%)
Urinary system	8(1.1%)	6(6.5%)	2(0.4%)	0(0%)
Multi-system	45(6.3%)	26(28.3%)	18(3.9%)	1(0.6%)

The percentage is the constituent ratio of total number or different triage group.

The clinical manifestations varied according to the type of toxic exposure. As mentioned above, gastrointestinal manifestation was common across all toxicants, especially in children with the exposed to pesticides (Supplementary Table S8). Relatively, skin and mucous membrane manifestation was more prevalent in children with corrosive household products exposure (11.4%), while respiratory system manifestation was primarily common in children with inhaled toxic gases exposure (21.6%). Nervous system manifestation was most commonly in children with alcohol-containing substances and plants/mushrooms exposure (16.7%). Multi-system manifestation was most frequently observed in cases with pesticide exposure (15.0%) (Supplementary Table S8).

### 3.4 Toxicology detection

In this study, during the patient evaluation process, if the physician identified a history of exposure to pesticides, highly toxic industrial products, or toxic heavy metals, or if the patient presented with significant multi-system manifestation, comprehensive toxicology testing was recommended. Upon obtaining parental consent, the necessary testing will be conducted. A total of 189 admitted children underwent toxicology testing, with a positive detection rate of 40.7%. There were significant differences in the rates of toxicology testing and the positive rate between the red zone and other zone. In the red zone, 51.1% of patients underwent toxicology testing (*n* = 47), with a positive detection rate of 74.5%. In the yellow zone, 28.1% of patients were tested (*n* = 130), with a positive detection rate of 29.2%. In the green zone, the positive detection rate was 33.3%. The distribution of positive toxicology results across different toxic categories is shown in Supplementary Table S9. The highest positive detection rates were observed for heavy metals (5/5, 100%), cosmetics (6/10, 60.0%), and alcohol-containing substances (6/11, 54.5%).

### 3.5 Clinical interventions and outcomes

**Figure 3:**
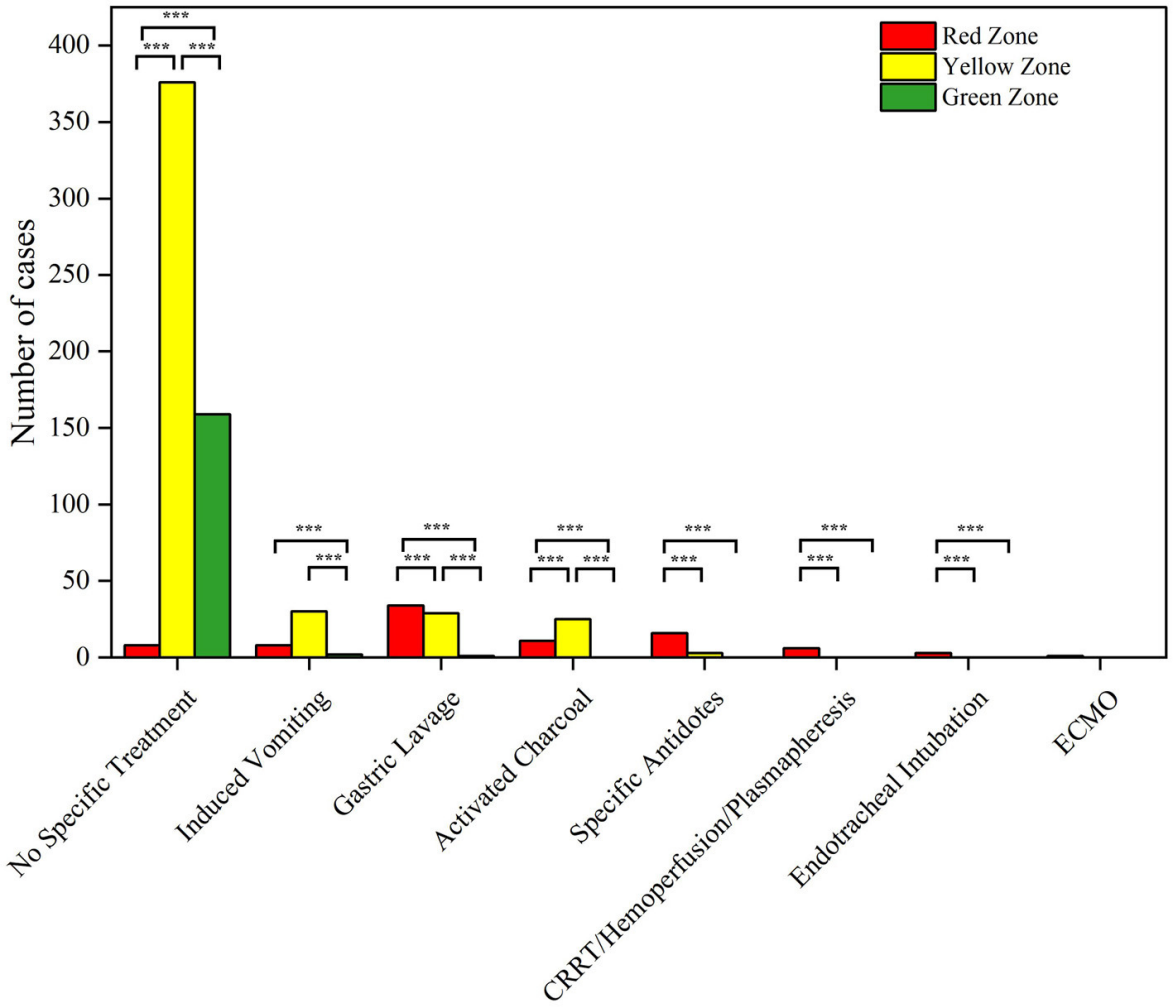
Pairwise comparison of clinical interventions in different triage age group (***P < 0.05).

Among children with acute non-pharmaceutical toxic exposures, 75.6% of them (*n* = 543) did not receive any specific treatment, especially for children triaged as green (97.5%). The proportion of patients in the red zone who received aggressive interventions such as gastric lavage, activated charcoal, specific antidotes, and advanced treatments [such as continuous renal replacement therapy (CRRT)/hemoperfusion/plasmapheresis] was significantly higher than those in the yellow and green zones (*P* < 0.05) (Figure 3). Particularly, endotracheal intubation and extracorporeal membrane oxygenation (ECMO) were used exclusively for red zone patients (Table 4, Supplementary Table S10). Additionally, poisoning cases who received antidote are summarized in Table 5.

**Table 4:** Clinical interventions and outcomes for children with acute non-pharmaceutical toxic exposures.

	**Total (*n =* 718)**	**Red zone (*n =* 92)**	**Yellow zone (*n =* 463)**	**Green zone (*n =* 163)**
**Clinical interventions**				
No specific treatment	543(75.6%)	8(8.7%)	376(81.2%)	159(97.5%)
Induced vomiting	40(5.6%)	8(8.7%)	30(6.5%)	2(1.2%)
Gastric lavage	64(8.9%)	34(37.0%)	29(6.3%)	1(0.6%)
Activated charcoal	36(5.0%)	11(12.0%)	25(5.4%)	0(0%)
Specific antidotes	19(2.6%)	16(17.4%)	3(0.6%)	0(0%)
CRRT/hemoperfusion/plasmapheresis	6(0.8%)	6(6.5%)	0(0%)	0(0%)
Endotracheal intubation	3(0.4%)	3(3.3%)	0(0%)	0(0%)
ECMO	1(0.1%)	1(1.1%)	0(0%)	0(0%)
**Outcomes**				
Go home after a short observation (< 6 h)	563(78.4%)	8(8.7%)	401(86.6%)	154(94.5%)
Emergency observation	137(19.1%)	66(71.7%)	62(13.4%)	9(5.5%)
Hospital admission	18(2.5%)	18(19.6%)	0(0%)	0(0%)

The percentage is the constituent ratio of total number or different triage group.

**Table 5:** Summary of poisoning cases receiving antidote therapy.

**Poisoning type**	**Antidote**	**Number of cases**
Organophosphate	Atropine	4
Carbamate		3
Lead	Edetate calcium disodium	2
Cyanide	Sodium nitrite and sodium thiosulfate	3
Hydrogen sulfide	Sodium nitrite	2
Sodium nitrite	Methylene blue	2
Hydrofluoric acid	Calcium gluconate	1
Carbon monoxide	Hyperbaric oxygen	2

In terms of clinical outcomes, 78.4% of children with acute non-pharmaceutical toxic exposures discharged after a short observation, with the highest proportion in the green zone (94.5%). 71.7% of patients in the red zone required emergency observation, while a smaller proportion of yellow zone patients required observation (13.4%). Hospital admissions only occurred in the red zone patients (19.6%) (Table 4).

### 3.6 Factors associating with observation/hospitalization

Univariate and multivariate binary logistic regression analysis of observation or hospitalization following acute non-pharmaceutical toxic exposure in children. In the univariate analysis, all extracted variables were tested (Table 6). The multivariate analysis confirmed that corrosive household products exposure (OR = 42.747, 95% CI [5.041–362.520]), skin and mucous membrane manifestation (OR = 37.052, 95% CI [5.339–257.153]), pesticides exposure (OR = 33.322, 95% CI [3.863–287.423]), heavy metals exposure (OR = 31.636, 95% CI [1.471–680.210]), neurological manifestation (OR = 22.656, 95% CI [4.766–107.711]), positive toxicology results (OR = 15.105, 95% CI [6.584–34.656]), industrial products exposure (OR = 10.294, 95% CI [1.144–92.641]), and intentional poisoning (OR = 3.060, 95% CI [1.733–5.405]) remained significant for observation/hospitalization (*P* < 0.05). Thus these factors might contribute to treatment decision-making process for pediatric acute non-pharmaceutical toxic exposure.

**Table 6:** Factors associating with observation/hospitalization after pediatric acute non-pharmaceutical exposures.

**Variable**	**Univariate analysis**		**Multivariate analysis**	
	**P**	**OR(95%CI)**	**P**	**OR(95%CI)**
Age 12–18 years	0.000	3.571 (1.868–6.827)	0.268	1.744 (0.650–4.677)
Industrial products exposure	0.16	12.500 (1.595–97.951)	0.038	10.294 (1.144–92.641)
Heavy Metals	0.001	34.615 (4.571–262.136)	0.027	31.636 (1.471–680.210)
Corrosive household products exposure	0.000	200.000 (10.437–3832.608)	0.001	42.747 (5.041–362.520)
Pesticides exposure	0.000	41.860 (5.507–318.214)	0.001	33.322 (3.863–287.423)
Skin and mucous membrane manifestation	0.000	41.228 (8.813–192.864)	0.000	37.052 (5.339–257.153)
Circulatory system manifestation	0.023	16.491 (1.472–184.744)	0.181	6.682 (0.415–107.699)
Nervous system manifestation	0.000	52.222 (14.987–181.963)	0.000	22.656 (4.766–107.711)
Hematologic system manifestation	0.006	24.737 (2.531–241.797)	0.297	4.133 (0.287–59.531)
Positive toxicology results	0.000	16.76 (9.643–29.129)	0.000	15.105 (6.584–34.656)
Intentional exposure	0.000	6.367 (4.131–9.813)	0.000	3.060 (1.733–5.405)

## 4 Discussion

Acute poisoning is one of the leading public health issues affecting children globally. Non-pharmaceutical poisonings accounting for as much as 53% to 67% in all pediatric acute exposure cases (13–15). This study aimed to examine the epidemiological characteristics, clinical presentations, treatment, and outcomes of non-pharmaceutical toxic exposures in children in our emergency department over the past 5 years. The findings can provide scientific evidence for the formulation of public health policies for optimizing the pediatric clinical practice guidelines, as well as enhance health education efforts to reduce the incidence of non-pharmaceutical toxic exposures.

The distribution of non-pharmaceutical poisoning cases varies across China. For example, in Eastern China, 2,952 cases of non-pharmaceutical poisoning were reported in Zhejiang from 2006 to 2015 (5), while 27 cases were documented in Shanghai over 2 years (6). In the Northeast China, 100 cases of non-pharmaceutical poisoning were reported in Jilin from 2016 to 2022 (7), and 507 cases were reported in Liaoning from 2012 to 2016 (8). In Southwest China, 381 cases were recorded in Chongqing between 2012 to 2020 (2). Our center recorded an average of 144 cases per year, indicating a medium occurrence rate. The highest proportion of cases occurred in infants and toddlers (< 3 years), with a slight higher gender ratio of male (1.16:1). Most exposures happened at home (89.3%), consistent with studies from other regions (16). Notably, 64.9% of intentional exposure patients were adolescent with depression. Lee et al. (10) and Parvin et al. (17) found that female adolescent are the primary population in the intentional poisoning events. Younger boys are more prone to accidental poisoning due to their active behavior, These highlighted the importance of tailored prevention strategies for different age and gender groups. For example, for infants and toddlers, enhancing the safety management and supervision of toxic substances in the home is essential; while for adolescents, particularly girls, there should be a focus on mental health education and intervention, with timely identification and management of potential psychological issues. For example, early screening in clinical practice and the implementation of cognitive-behavioral therapy (CBT) (18) for intervention; collaboration between schools and families to complete mental health education for high-risk groups; and multidisciplinary cooperation to ensure comprehensive solutions for children with mental health issues.

Most exposures occurred in the afternoon and evening (16:00 to 24:00, accounting for 52.1%), which may be related to increased opportunities for children to encounter toxic substances after school. Seasonal trends showed a higher incidence in autumn, with pesticides being the most common toxicant, especially in rural areas. The incidence of pesticide poisonings has been on the rise due to agricultural activities, particularly in summer (5, 19). Additionally, the prevalence of infectious diseases has led to increased use of mercury thermometers, which was the most frequent cause of poisoning identified in our study. Consist with previous studies (20, 21), oral ingestion was the primary route of poisoning (93.6%), such phenomenon is closely related to children's tendency to put objects in their mouths. The study showed that the primary caregivers in non-pharmaceutical toxic exposure incidents were mainly parents (51.4%), followed by grandparents (25.6%). Despite the high educational level of caregivers (50.7% with a college/university degree), the incidence of poisoning remains concerning. Therefore, it is essential to protect children's safety through multiple channels, such as helping them understand the dangers of ingesting foreign substances and offering sufficient train for their caregivers.

Notably, the proportion of toxic exposure cases peaked in 2020, but decreased in 2023. Several studies have also reported an increase in poisoning cases during the COVID-19 pandemic (22–28). Children spending more time at home due to quarantine may face greater exposure to household toxins. Additionally, the excessive use of sanitizers driven by anxiety about COVID-19 infection leads to an increase in poisoning incidents. At the same time, an increase in intentional exposures has been linked to mental health issues exacerbated by quarantine measures. This trend during public health crises highlights the critical need to strengthen household poison management and enhance child safety education. Sustained attention and the continued optimization of these preventive strategies are essential to reducing the occurrence of acute toxic exposures in children moving forward.

Mercury was the most common non-pharmaceutical exposure substance in this cohort, it is mainly due to mercury exposure from broken thermometers. Although mercury exposure was prevalent, it typically poses minimal health risk under short-term exposure (29). Apart from mercury, pesticides were the most common toxicant and present a more significant concern, it accounted for 17.7% and were more frequent in rural areas. Therefore, parents should be particularly cautious about the storage locations of these hazardous items, especially in rural households, ensuring they are kept out of children's reach. The study also found that exposure to corrosive household cleaning products was relatively high, occurring across all age groups, but seemed higher among adolescents. Many studies have shown that some adolescents intentionally come into contact with these substances, often linked to depression or emotional distress (30).

Previous studies have shown that clinical symptom in pediatric acute poisoning cases often manifests as asymptomatic or mildly symptomatic, with laboratory tests usually showing no abnormalities (31, 32). Our findings are consistent with this, as 77.6% of children with acute non-pharmaceutical exposures exhibited major symptoms after poisoning, with 75.6% not requiring specialized treatment. This is consistent with existing research findings (16, 33, 34), and may raise concerns about resource utilization, prompting the question of whether many of these children truly need to visit the hospital. A consultation platform for pediatric poisonings to provide initial remote guidance might be beneficial to solve this problem (21). However, some patients with critical condition indeed require timely treatment. Compared to patients in the green and yellow zones, those in the red zone had a significantly higher proportion of clinical symptom and a markedly increased incidence of multi-system manifestation, leading to more frequent clinical interventions. These results align with the principles of emergency triage and suggest that patients in the red zone require more urgent and comprehensive medical interventions, advanced life support technique was crucial for some patients with severe status (35). Additionally, 51.1% of red zone patients underwent toxicology testing, with toxic substances detected in 38% of cases. In contrast, only 28.1% of yellow zone patients were tested, with a positive rate of 8.2%, and almost no testing was conducted in the green zone. These findings indicate that patients in the red zone, due to their more severe clinical presentations, require toxicology testing more urgently, with a higher likelihood of positive results. Combing with comprehensive evaluation based on the patient's clinical presentation, toxic exposure details, and toxicology test results, appropriate triage can be made to guide following disposition by physician.

In terms of outcomes, 78.4% of patients were discharged directly, with the highest proportion seen in the green zone (94.5%). However, among red zone patients, 71.7% required emergency observation, and 19.6% needed hospitalization. The severity of toxic exposure directly impacted prognosis. Proper triage, along with tailored treatment based on the patient's clinical presentation and laboratory findings, can help optimize the allocation of medical resources and enhance the efficiency of emergency care.

The logistic regression analysis revealed that corrosive household products exposure, skin and mucous membrane manifestation, pesticides exposure, heavy metals exposure, neurological manifestation, positive toxicology results, industrial products exposure, and intentional poisoning were significant factors associated with the need for emergency observation or hospitalization. A variety of poisons can influence patient outcomes (35). Our findings indicate that many cases requiring observation or hospitalization were due to household items commonly used in daily life. Symptomatology is another critical factor impacting the outcome of poisoning cases. Neurological symptoms are a known risk factor for an increased PSS (36, 37), and patients exhibiting such symptoms demand heightened attention from medical providers. Suicide is the primary cause for intentional poisoning cases (38) and patients with mental health may use multiple or higher amount of toxicants, which can lead to serious consequences. The association between poisoning and a positive toxicology result was identified for the first time in our study, emphasizing the importance of obtaining such results promptly. With clear signs of poisoning, clinicians must act quickly to initiate treatment.

To reduce the incidence of pediatric acute toxic exposures, we must address the root cause: improper management of household toxicants. Raising awareness among families and society about the dangers of toxic substances to prevent childhood poisonings. Firstly, strengthening parents' and caregivers' ability to manage toxic substances—particularly the safe storage of cleaning agents, pesticides, and thermometer—can be achieved through targeted initiatives such as community activities, hospital-led education sessions, and school programs. Secondly, leveraging media and social platforms to disseminate poison prevention knowledge is essential. These efforts should emphasize the importance of storing toxic substances out of children's reach and in child-resistant containers. Thirdly, government action is vital. This includes enacting stringent chemical management regulations, strengthening market supervision, and ensuring that products meet safety standards. Establishing a national poison control center offering 24-h emergency consultation services would be a significant step forward. Public health initiatives, such as promoting “Family Safety Month,” could also play a pivotal role in raising widespread awareness of poison prevention strategies. By strengthening poison prevention and management efforts through the combined efforts of families, communities, schools, and the government, the incidence of pediatric acute toxic exposures can be significantly reduced, ensuring the health and safety of children.

Despite providing valuable insights, this study has certain limitations. First, as a single-center study, the generalizability of its findings may be limited, necessitating further validation across different regions and medical institutions. Second, this study did not investigate the long-term health effects of specific toxic exposures. While poisoning cases—whether or not they resulted in observation or hospitalization—were set as outcomes in the logistic regression analysis, identifying additional risk factors associated with these outcomes could be valuable for future triage strategies. Third, the results derived from the multiple logistic regression analysis should be interpreted with caution. The small subgroup sample size led to wide confidence intervals for several predictors, and potential overfit between different predictor categories may introduce bias when applying these risk factors in clinical practice. To address these limitations, future research should focus on multi-center, large-sample prospective studies to validate and expand upon the findings of this study. Such studies should also aim to explore the long-term health effects of various toxic exposures to enhance our understanding and improve patient care.

## 5 Conclusion

This study revealed the epidemiological characteristics of pediatric acute non-pharmaceutical toxic exposures and their impact on clinical management, emphasizing the importance of some specific toxicants management and child safety education. Implementing these measures might effectively reduce the occurrence of toxic exposure incidents, thereby better protecting children's health and safety.

## Data Availability

The raw data supporting the conclusions of this article will be made available in a deidentified form by the corresponding authors, without undue reservation.
