# Supplementary material for: Analysis of acute non-pharmaceutical toxic exposures in children: a 5-year retrospective study
Source: Front Public Health. 2025 Feb 5;13:1510205. doi: 10.3389/fpubh.2025.1510205 (PMC11836825; doi:10.3389/fpubh.2025.1510205)
Supplement: Supplementary file 1 [file Supplementary_file_1.pdf]

Table S1. The age groups and gender distribution within the study population

	Infant and Toddler	Preschool age	School age	Adolescence	Total
Male	219(52.6%)	86(51.5%)	59(63.4%)	21(50.0%)	385(53.6%)
Female	197(47.4%)	81(48.5%)	34(36.6%)	21(50.0%)	333(46.4%)

Table S2. Types of non-pharmaceutical exposure stratified by age

N(%)	Total (n=718)	Infant and Toddler (n=416)	Preschool age (n=167)	School age (n=93)	Adolescence (n=42)
**Mercury**	135(18.8%)	67(16.1%)	53(31.7%)	14(15.1%)	1(2.4%)
**Pesticides**	127(17.7%)	92(22.1%)	21(12.6%)	6(6.5%)	8(19.0%)
**Corrosive Household Products**	88(12.3%)	45(10.8%)	21(12.6%)	11(11.8%)	11(26.2%)
**Non-Corrosive Household Products**	76(10.6%)	40(9.6%)	16(9.6%)	13(14.0%)	7(16.7%)
**Industrial Products**	75(10.4%)	30(7.2%)	25(15.0%)	14(15.1%)	6(14.3%)
**Desiccants**	73(10.2%)	51(12.3%)	10(6.0%)	10(10.8%)	2(4.8%)
**Cosmetics**	51(7.1%)	42(10.1%)	5(3.0%)	3(3.2%)	1(2.4%)
**Alcoholic Products**	30(4.2%)	17(4.1%)	6(3.6%)	4(4.3%)	3(7.1%)
**Paints and Inks**	26(3.6%)	14(3.4%)	4(2.4%)	8(8.6%)	0(0%)
**Plants/Mushrooms**	18(2.5%)	11(2.6%)	2(1.2%)	4(4.3%)	1(2.4%)
**Contaminated Food**	14(1.9%)	4(1.0%)	4(2.4%)	6(6.5%)	0(0.0%)
**Heavy Metals**	5(0.7%)	3(0.7%)	0(0%)	0(0%)	2(4.8%)

**Inhaled Toxic Gases**	37(5.2%)	22(5.3%)	9(5.4%)	5(5.3%)	1(2.4%)

Note： The percentage is the constituent ratio of total number or different age group.

Table S3. Details of exposure causes in different poisoning category

Poisoning category	Details
**Pesticides (n=127)**	Organophosphorus pesticide (n=16, 12.6%)<br>Carbamates pesticide (n=48, 37.8%)<br>Pyrethriods pesticide (n=35, 27.6%)<br>Chlorinated hydrocarbon pesticides (n=7, 5.5%)<br>Anticoagulant rodenticide (n=11, 8.7%)<br>Neurotoxic rodenticide (n=6, 4.7%)<br>Others (n=4, 3.1%)
**Corrosive Household Products (n=88)**	Chlorine bleach (n=31, 35.2%)<br>Detergent containing ammonia (n=15, 17.0%)<br>Acid detergent (n=5, 5.7%)<br>Disinfectant(containing chlorine or hydrogen peroxide) (n=12, 13.6%)<br>Oil detergent (n=10, 11.4%)<br>Drain cleaner (n=8, 9.1%)<br>Others (n=7, 8.0%)
**Non-Corrosive Household Products (n=76)**	Surfactant-based detergents (n=29, 38.2%)<br>Benzene and aldehyde detergent (n=9, 11.8%)<br>Laundry detergent (n=20, 26.3%)<br>Air freshener (n=7, 9.3%)

	Iodophor disinfectant (n=8, 10.5%)<br>Others (n=3, 3.9%)
**Industrial Products (n=75)**	Organic solvents (n=21, 28.0%)<br>Plasticizer (n=14, 18.6%)<br>Paint coating (n=12, 16.0%)<br>Gasoline (n=9, 12.0%)<br>Cyanide (n=5, 6.7%)<br>Toxic dyes (n=4, 5.3%)<br>Nitrite (n=3, 4.0%)<br>Others (n=7, 9.3%)
**Inhaled Toxic Gases (n=37)**	Carbon monoxide (n=18, 48.6%)<br>Methane (n=6, 16.2%)<br>Ammonia gas (n=5, 13.5%)<br>Sulfureted hydrogen (n=3, 8.1%)<br>Chlorine (n=3, 8.1%)<br>Phosphine (n=2, 5.5%)

Table S4. Time period of treatment for patients with different exposure types

	**Mercury (n=135)**	**Pesticides (n=127)**	**Corrosive Household Products (n=88)**	**Non-Corrosive Household Products (n=76)**	**Industrial Products (n=38)**	**Desiccants (n=73)**	**Cosmetics (n=51)**	**Alcoholic Products (n=30)**	**Paints and Inks (n=26)**	**Plants/Mushrooms (n=14)**	**Contaminated Food (n=5)**	**Inhaled Toxic Gases (n=37)**
**8:00-16:00**	47(34.8%)	66(52.0%)	36(40.9%)	22(28.9%)	12(31.6%)	30(41.1%)	23(45.1%)	5(16.7%)	10(38.5%)	8(44.5%)	1(20.0%)	14(37.8%)
**16:00-24:00**	70(53.3%)	51(40.1%)	43(48.9%)	45(59.2%)	23(60.5%)	42(57.5%)	25(49.0%)	21(70.0%)	14(53.8%)	10(55.5%)	2(60.0%)	20(54.1%)
**0:00-8:00**	18(11.9%)	10(7.9%)	9(10.2%)	9(11.8%)	3(7.9%)	1(1.4%)	3(5.9%)	4(13.3%)	2(7.7%)	0(0%)	1(20.0%)	3(8.1%)

Table S5. Pairwise comparison of different age group for non-pharmaceutical exposure

Age Group	Drug Comparison	P-value	Age Group	Drug Comparison	P -value	Age Group	Drug Comparison	P -value
Mercury	Infant and Toddler *vs.* Preschool age	4.6166E-05	Pesticides	Infant and Toddler *vs.* Preschool age	0.0121	Corrosive Household Products	Infant and Toddler *vs.* Preschool age	0.6486
	Infant and Toddler *vs.* School age	0.9230		Infant and Toddler *vs.* School age	0.0010		Infant and Toddler *vs.* School age	0.9240
	Infant and Toddler *vs.* Adolescence	0.0360		Infant and Toddler *vs.* Adolescence	0.8625		Infant and Toddler *vs.* Adolescence	0.0062
	Preschool age *vs.* School age	0.0055		Preschool age *vs.* School age	0.1819		Preschool age *vs.* School age	1
	Preschool age *vs.* Adolescence	0.0003		Preschool age *vs.* Adolescence	0.3647		Preschool age *vs.* Adolescence	0.0422
	School age *vs.* Adolescence	0.0381*		School age *vs.* Adolescence	0.0320*		School age *vs.* Adolescence	0.0560
Non-Corrosive Household Products	Infant and Toddler *vs.* Preschool age	1	Industrial Products	Infant and Toddler *vs.* Preschool age	0.0063	Desiccants	Infant and Toddler *vs.* Preschool age	0.0371
	Infant and Toddler *vs.* School age	0.2929		Infant and Toddler *vs.* School age	0.0263		Infant and Toddler *vs.* School age	0.8183
	Infant and Toddler *vs.* Adolescence	0.1713		Infant and Toddler *vs.* Adolescence	0.1195*		Infant and Toddler *vs.* Adolescence	0.2065*
	Preschool age *vs.* School age	0.3833		Preschool age *vs.* School age	1		Preschool age *vs.* School age	0.2555
	Preschool age *vs.* Adolescence	0.1723*		Preschool age *vs.* Adolescence	1		Preschool age *vs.* Adolescence	1*

	School age *vs.* Adolescence	0.8336		School age *vs.* Adolescence	1		School age *vs.* Adolescence	0.3452*
Cosmetics	Infant and Toddler *vs.* Preschool age	0.0074	Alcoholic Products	Infant and Toddler *vs.* Preschool age	0.9652	Paints and Inks	Infant and Toddler *vs.* Preschool age	0.7271
	Infant and Toddler *vs.* School age	0.0568		Infant and Toddler *vs.* School age	1*		Infant and Toddler *vs.* School age	0.0426*
	Infant and Toddler *vs.* Adolescence	0.1587*		Infant and Toddler *vs.* Adolescence	0.4090*		Infant and Toddler *vs.* Adolescence	0.6254*
	Preschool age *vs.* School age	1*		Preschool age *vs.* School age	0.7487*		Preschool age *vs.* School age	0.0307*
	Preschool age *vs.* Adolescence	1*		Preschool age *vs.* Adolescence	0.3837*		Preschool age *vs.* Adolescence	1*
	School age *vs.* Adolescence	1*		School age *vs.* Adolescence	0.4360*		School age *vs.* Adolescence	0.1062*
Plants/Mushrooms	Infant and Toddler *vs.* Preschool age	0.3670*	Contaminated Food	Infant and Toddler *vs.* Preschool age	0.2350*	Heavy Metal	Infant and Toddler *vs.* Preschool age	0.5613*
	Infant and Toddler *vs.* School age	0.4936*		Infant and Toddler *vs.* School age	0.0036*		Infant and Toddler *vs.* School age	1*
	Infant and Toddler *vs.* Adolescence	1*		Infant and Toddler *vs.* Adolescence	1*		Infant and Toddler *vs.* Adolescence	0.0657*
	Preschool age *vs.* School age	0.1915*		Preschool age *vs.* School age	0.1749*		Preschool age *vs.* School age	1*
	Preschool age *vs.* Adolescence	0.4827*		Preschool age *vs.* Adolescence	1*		Preschool age *vs.* Adolescence	0.0378*
	School age *vs.* Adolescence	1*		School age *vs.* Adolescence	0.1775*		School age *vs.* Adolescence	0.0915*

Inhaled Toxic Gases	Infant and Toddler *vs.* Preschool age	1						
	Infant and Toddler *vs.* School age	1*						
	Infant and Toddler *vs.* Adolescence	0.7103*						
	Preschool age *vs.* School age	1						
	Preschool age *vs.* Adolescence	0.6909*						
	School age *vs.* Adolescence	0.6671*						

*: Fisher test.

Table S6. Pairwise comparison of different zone for clinical manifestation

Manifestation	Zone Comparison	P-value	Manifestation	Zone Comparison	P-value	Manifestation	Zone Comparison	P-value
No Manifestation	Red Zone *vs.* Yellow Zone	2.9019E-29	Gastrointestinal	Red Zone *vs.* Yellow Zone	0.0616	Skin or Mucous	Red Zone *vs.* Yellow Zone	7.1109E-05*
	Red Zone *vs.* Green Zone	1.8707E-36		Red Zone *vs.* Green Zone	6.0821E-05		Red Zone *vs.* Green Zone	0.0033
	Yellow Zone *vs.* Green Zone	3.9737E-07		Yellow Zone *vs.* Green Zone	0.0075		Yellow Zone *vs.* Green Zone	1*
Respiratory	Red Zone *vs.* Yellow Zone	0.3801*	Nervous	Red Zone *vs.* Yellow Zone	0.0019	Circulatory	Red Zone *vs.* Yellow Zone	4.6176E-06
	Red Zone *vs.* Green Zone	0.0262*		Red Zone *vs.* Green Zone	1.3859E-05*		Red Zone *vs.* Green Zone	6.4534E-05*

	Yellow Zone *vs.* Green Zone	0.0752*		Yellow Zone *vs.* Green Zone	0.0174*		Yellow Zone *vs.* Green Zone	0.5775*
Hematologic	Red Zone *vs.* Yellow Zone	0.0053*	Urinary	Red Zone *vs.* Yellow Zone	0.0024*	Multi-system	Red Zone *vs.* Yellow Zone	9.6847E-08*
	Red Zone *vs.* Green Zone	0.0028*		Red Zone *vs.* Green Zone	0.0125*		Red Zone *vs.* Green Zone	6.3446E-08*
	Yellow Zone *vs.* Green Zone	0.3444*		Yellow Zone *vs.* Green Zone	1*		Yellow Zone *vs.* Green Zone	0.0572*

*: Fisher test.

Table S7. Clinical manifestations of children with acute non-pharmaceutical toxic exposures

		**Total (n=718)**	**Red Zone (n=92)**	**Yellow Zone (n=463)**	**Green Zone (n=163)**
**No symptom**		557(77.6%)	32(34.8%)	375(81.0%)	150(92.0%)
**Gastrointestinal System**	**Nausea and vomiting**	107(14.9%)	32(34.8%)	65(14.0%)	10(6.1%)
	**Abdominal pain and diarrhea**	33(4.6%)	7(7.6%)	23(5.0%)	3(1.8%)
	**Acute liver injury**	8(1.1%)	7(7.6%)	1(0.2%)	0(0%)
**Skin or Mucous Membrane**	**Burning sensation of oral mucosa**	12(1.7%)	10(10.9%)	1(0.2%)	1(0.6%)
	**Cutaneous mucosal injury**	7(1.0%)	2(2.2%)	5(1.1%)	0(0%)
**Respiratory System**	**Cough or sore throat**	13(1.8%)	2(2.2%)	11(2.4%)	0(0%)
	**Dyspnea**	3(0.4%)	3(3.3%)	0(0%)	0(0%)

**Nervous System**	**Excitement or delirium**	4(0.6%)	1(1.1%)	3(0.6%)	0(0%)
	**Dizziness and headache**	26(3.6%)	12(13.0%)	14(3.0%)	0(0%)
	**Convulsion**	3(0.4%)	3(3.3%)	0(0%)	0(0%)
	**Disturbance of consciousness**	2(0.3%)	2(2.2%)	0(0%)	0(0%)
**Circulatory System**	**Panic and fatigue**	9（1.3%）	5(5.4%)	4(0.9%)	0(0%)
	**Pale and sweat**	5(0.7%)	5(5.4%)	0(0%)	0(0%)
	**Hypotension**	3(0.4%)	3(3.3%)	0(0%)	0(0%)
**Hematologic System**	**Abnormal coagulation function**	14(1.9%)	8(8.7%)	6(1.3%)	0(0%)
**Urinary System**	**Hematuria or proteinuria**	6(0.8%)	4(4.3%)	2(0.4%)	0(0%)
	**Acute kidney injury**	4(0.6%)	4(4.3%)	0(0%)	0(0%)
**Multi-System**		45(6.3%)	26(28.3%)	18(3.9%)	1(0.6%)

Note: Multiple manifestations might occurred at single patients.

Table S8. Distribution of clinical manifestation by non-pharmaceutical exposure categories

Clinical Manifestation	Mercury (n=135)	Pesticides (n=127)	Corrosive Household Products (n=88)	Non-Corrosive Household Products (n=76)	Industrial Products (n=75)	Desiccants (n=73)	Cosmetics (n=51)	Alcoholic Products (n=30)	Paints and Inks (n=26)	Plants/Mushrooms (n=18)	Contaminated Food (n=14)	Heavy Metals (n=5)	Inhaled Toxic Gases (n=37)
**No Manifestation**	133(98.5%)	66(52.0%)	56(63.6%)	57(75.0%)	47(62.7%)	66(90.4%)	43(84.3%)	17(56.7%)	22(84.6%)	14(77.8%)	10(71.4%)	4(80.0%)	22(59.5%)
**Gastrointestinal System**	2(1.5%)	46(36.2%)	26(29.5%)	19(25.0%)	13(17.4%)	7(9.6%)	8(15.7%)	7(23.3%)	4(15.4%)	3(16.7%)	3(21.4%)	1(0.0%)	1(2.7%)

**Skin or Mucous Membrane**	0(0.0%)	5(3.9%)	10(11.4%)	0(0.0%)	4(5.3%)	0(0.0%)	0(0.0%)	0(0.0%)	0(0.0%)	0(0.0%)	0(0.0%)	0(0.0%)	0(0.0%)
**Respiratory System**	0(0.0%)	0(0%)	4(4.5%)	0(0.0%)	4(5.3%)	0(0.0%)	0(0.0%)	0(0.0%)	0(0.0%)	0(0.0%)	0(0.0%)	0(0.0%)	8(21.6%)
**Nervous System**	0(0.0%)	12(9.4%)	0(0%)	0(0.0%)	8(10.7%)	0(0.0%)	0(0.0%)	5(16.7%)	0(0.0%)	3(16.7%)	2(14.3%)	0(0.0%)	2(5.4%)
**Circulatory System**	0(0.0%)	8(6.3%)	0(0%)	0(0.0%)	3(4.0%)	0(0.0%)	0(0.0%)	2(6.7%)	0(0.0%)	0(0.0%)	1(7.1%)	0(0.0%)	3(8.1%)
**Hematologic System**	0(0.0%)	8(6.3%)	0(0%)	0(0.0%)	2(2.7%)	0(0.0%)	0(0.0%)	2(6.7%)	0(0.0%)	0(0.0%)	0(0.0%)	0(0.0%)	2(5.4%)
**Urinary System**	0(0.0%)	4(3.1%)	0(0.0%)	0(0.0%)	1(1.3%)	0(0.0%)	0(0.0%)	0(0.0%)	0(0.0%)	0(0.0%)	0(0.0%)	0(0.0%)	3(8.1%)
**Multi-System Involvement**	0(0.0%)	19(15.0%)	8(9.1%)	0(0%)	7(9.3%)	0(0.0%)	0(0.0%)	3(10.0%)	0(0.0%)	2(11.1%)	2(14.3%)	0(0.0%)	4(10.8%)

Note: The percentage is the constituent ratio of different exposure group.

Table S9. Distribution of positive toxicology results by toxicant categories

	**Mercury (n=1)**	**Pesticides (n=60)**	**Corrosive Household Products (n=20)**	**Non-Corrosive Household Products (n=25)**	**Industrial Products (n=35)**	**Desiccants (n=10)**	**Cosmetics (n=10)**	**Alcoholic Products (n=11)**	**Paints and Inks (n=2)**	**Plants/Mushrooms (n=7)**	**Contaminated Food (n=3)**	**Heavy Metals (n=5)**
**Positive Toxicology Results**	0(0.0%)	28(46.7%)	6(30.0%)	13(52.0%)	10(28.6%)	1(10.0%)	6(60.0%)	6(54.5%)	0(0%)	1(14.3%)	1(33.3%)	5(100%)

Table S10. Pairwise comparison of different zone for clinical intervention

**Clinical interventions**	**Zone Comparison**	**P-value**	**Clinical interventions**	**Zone Comparison**	**P-value**	**Clinical interventions**	**Zone Comparison**	**P-value**
No Specific Treatment	Red Zone *vs.* Yellow Zone	2.3714E-40	Induced Vomiting	Red Zone *vs.* Yellow Zone	0.4926	Gastric Lavage	Red Zone *vs.* Yellow Zone	5.8700E-14
	Red Zone *vs.* Green Zone	3.8726E-51		Red Zone *vs.* Green Zone	0.0040*		Red Zone *vs.* Green Zone	4.1366E-17
	Yellow Zone *vs.* Green Zone	2.5324E-07		Yellow Zone *vs.* Green Zone	0.0164		Yellow Zone *vs.* Green Zone	0.0074
Activated Charcoal	Red Zone *vs.* Yellow	0.0232	Specific Antidotes	Red Zone *vs.*	2.8571E-11*	CRRT/Hemoperfusion	Red Zone *vs.*	1.3499E-05*

	Zone			Yellow Zone		/Plasmapheresis	Yellow Zone	
	Red Zone *vs.* Green Zone	6.1307E-06*		Red Zone *vs.* Green Zone	1.8567E-08*		Red Zone *vs.* Green Zone	0.0016*
	Yellow Zone *vs.* Green Zone	0.0007*		Yellow Zone *vs.* Green Zone	0.5721*		Yellow Zone *vs.* Green Zone	1*
Endotracheal Intubation	Red Zone *vs.* Yellow Zone	0.0038*	ECMO	Red Zone *vs.* Yellow Zone	0.1582*			
	Red Zone *vs.* Green Zone	0.0417*		Red Zone *vs.* Green Zone	0.3494*			
	Yellow Zone *vs.* Green Zone	1*		Yellow Zone *vs.* Green Zone	1*			

*: Fisher test.
